# 2-Chloro-*N*-(4-methyl­benzo­yl)benzene­sulfonamide

**DOI:** 10.1107/S1600536810018908

**Published:** 2010-05-26

**Authors:** B. Thimme Gowda, Sabine Foro, P. A. Suchetan, Hartmut Fuess

**Affiliations:** a Department of Chemistry, Mangalore University, Mangalagangotri 574 199, Mangalore, India; b Institute of Materials Science, Darmstadt University of Technology, Petersenstrasse 23, D-64287 Darmstadt, Germany

## Abstract

In the title compound, C_14_H_12_ClNO_3_S, the conformation of the N—H bond in the C—SO_2_—NH—C(O) segment is *anti* to the C=O bond. The dihedral angle between the sulfonyl benzene ring and the —SO_2_—NH—C—O segment is 89.4 (1)° and that between the sulfonyl and benzoyl benzene rings is 89.1 (2)°. The crystal structure features inversion-related dimers linked by pairs of N—H⋯O hydrogen bonds.

## Related literature

For background to our study of the effect of ring and side-chain substituents on the crystal structures of *N*-aromatic sulfonamides and for similar structures, see: Gowda *et al.* (2010**a* ▶,b* ▶); Suchetan *et al.* (2010**a* ▶,b* ▶).
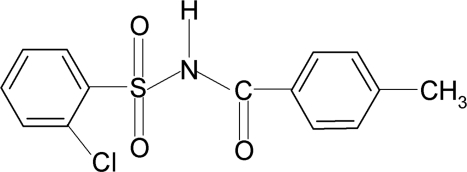


## Experimental

### 

#### Crystal data

C_14_H_12_ClNO_3_S
*M* *_r_* = 309.76
Monoclinic, 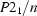

*a* = 8.0554 (8) Å
*b* = 23.209 (2) Å
*c* = 8.1199 (9) Å
β = 103.52 (1)°
*V* = 1476.0 (3) Å^3^
*Z* = 4
Mo *K*α radiation
μ = 0.41 mm^−1^
*T* = 299 K
0.40 × 0.30 × 0.25 mm

#### Data collection

Oxford Diffraction Xcalibur diffractometer with a Sapphire CCD detector
Absorption correction: multi-scan (*CrysAlis RED*; Oxford Diffraction, 2009 ▶) *T*_min_ = 0.855, *T*_max_ = 0.906
6106 measured reflections
3012 independent reflections
2396 reflections with *I* > 2σ(*I*)
*R*_int_ = 0.012

#### Refinement

*R*[*F* ^2^ > 2σ(*F* ^2^)] = 0.058
*wR*(*F* ^2^) = 0.153
*S* = 1.07
3012 reflections
184 parameters
19 restraints
H atoms treated by a mixture of independent and constrained refinement
Δρ_max_ = 0.43 e Å^−3^
Δρ_min_ = −0.54 e Å^−3^

### 

Data collection: *CrysAlis CCD* (Oxford Diffraction, 2009 ▶); cell refinement: *CrysAlis RED* (Oxford Diffraction, 2009 ▶); data reduction: *CrysAlis RED*; program(s) used to solve structure: *SHELXS97* (Sheldrick, 2008 ▶); program(s) used to refine structure: *SHELXL97* (Sheldrick, 2008 ▶); molecular graphics: *PLATON* (Spek, 2009 ▶); software used to prepare material for publication: *SHELXL97*.

## Supplementary Material

Crystal structure: contains datablocks I, global. DOI: 10.1107/S1600536810018908/bq2212sup1.cif

Structure factors: contains datablocks I. DOI: 10.1107/S1600536810018908/bq2212Isup2.hkl

Additional supplementary materials:  crystallographic information; 3D view; checkCIF report

## Figures and Tables

**Table 1:** Hydrogen-bond geometry (Å, °)

*D*—H⋯*A*	*D*—H	H⋯*A*	*D*⋯*A*	*D*—H⋯*A*
N1—H1*N*⋯O2^i^	0.84 (2)	2.14 (2)	2.970 (4)	169 (4)

Symmetry code: (i) 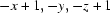.

**Fig. 1.**
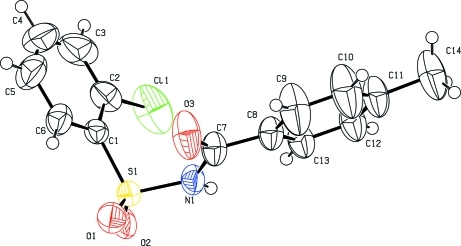
Molecular structure of the title compound, showing the atom-labelling scheme. Displacement ellipsoids are drawn at the 50% probability level.

**Fig. 2.**
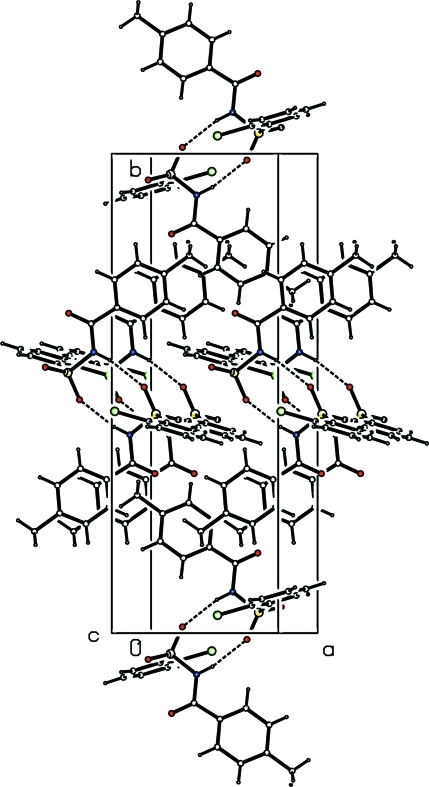
Molecular packing in the title compound. Hydrogen bonds are shown as dashed lines.

## supplementary crystallographic information

### Comment

Diaryl acylsulfonamides are known as potent antitumor agents against a broad spectrum of human tumor xenografts in nude mice. As a part of studying the effect of ring and the side chain substituents on the crystal structures of *N*-aromatic sulfonamides (Gowda *et al.*, 2010*a,b*; Suchetan *et al.*, 2010*a,b*), the structure of 2-chloro-*N*-(4-methylbenzoyl)benzenesulfonamide (I) has been determined (Fig.1). The conformations of the N—C bonds in the C—SO_2_—NH—C(O) segments have *gauche* torsions with respect to the SO bonds. Further, the conformation of the N—H bond in the C—SO_2_—NH—C(O) segment is *anti* to the C=O bond, similar to that observed in 2-methyl-*N*-(4-methylbenzoyl)-benzenesulfonamide (II) (Gowda *et al.*, 2010*a*), 2-chloro-*N*-(3-methylbenzoyl)-benzenesulfonamide (III) (Suchetan *et al.*, 2010*b*), 2-chloro-*N*-(2-methylbenzoyl)- benzenesulfonamide (IV) (Suchetan *et al.*, 2010*a*) and 2-chloro-*N*-(benzoyl)-benzenesulfonamide (V) (Gowda *et al.*, 2010*b*).

The molecules are twisted at the *S* atom with the torsional angle of 60.4 (3)°, compared to those of -53.1 (2)° and 61.2 (2)°, in the two molecules of (II), -66.5 (2)° in (III), -64.0 (2)° in (IV) and 66.7 (2)° in (V). The dihedral angle between the sulfonyl benzene ring and the —SO_2_—NH—C—O segment is 89.4 (1)°, compared to the values of 86.0 (1)° and 87.9 (1)° in the 2 molecules of (II), 88.4 (1)° in (III), 84.8 (1)° in (IV) and 87.3 (1)° in (V).

Furthermore, the dihedral angle between the sulfonyl and the benzoyl benzene ring is 89.1 (2)°, compared to the values of 88.1 (1)° (molecule 1) and 83.5 (1)° (molecule 2) of (II), 74.7 (1)° in (III), 78.7 (1)° in (IV) and 73.3 (1)° in (V).

The packing of molecules linked by of N—H···O(S) hydrogen bonds (Table 1) is shown in Fig. 2.

### Experimental

The title compound was prepared by refluxing a mixture of 4-methylbenzoic acid, 2-chlorobenzenesulfonamide and phosphorous oxy chloride for 5 h on a water bath. The resultant mixture was cooled and poured into ice cold water. The solid, 2-chloro-*N*-(4-methylbenzoyl)benzenesulfonamide obtained was filtered, washed thoroughly with water and then dissolved in sodium bicarbonate solution. The compound was later reprecipitated by acidifying the filtered solution with dilute HCl. The filtered and dried compound was recrystallized to the constant melting point.

Prism like colorless single crystals of the title compound used in X-ray diffraction studies were grown from a slow evaporation of its toluene solution at room temperature.

### Refinement

The H atoms of the NH groups were located in a difference map and later restrained to N—H = 0.86 (2) %A. The other H atoms were positioned with idealized geometry using a riding model with C—H = 0.93–0.96 Å. All H atoms were refined with isotropic displacement parameters (set to 1.2 times of the *U*_eq_ of the parent atom). The U^ij^ components of C4, C9 and C10 atoms were restrained to approximate isotropic behavior.

### Crystal data

**Table:**

C_14_H_12_ClNO_3_S	*F*(000) = 640
*M**_r_* = 309.76	*D*_x_ = 1.394 Mg m^−^^3^
Monoclinic, *P*2_1_/*n*	Mo *K*α radiation, λ = 0.71073 Å
Hall symbol: -P 2yn	Cell parameters from 2713 reflections
*a* = 8.0554 (8) Å	θ = 2.6–27.7°
*b* = 23.209 (2) Å	µ = 0.41 mm^−^^1^
*c* = 8.1199 (9) Å	*T* = 299 K
β = 103.52 (1)°	Prism, colourless
*V* = 1476.0 (3) Å^3^	0.40 × 0.30 × 0.25 mm
*Z* = 4	

### Data collection

**Table:**

Oxford Diffraction Xcalibur diffractometer with a Sapphire CCD detector	3012 independent reflections
Radiation source: fine-focus sealed tube	2396 reflections with *I* > 2σ(*I*)
graphite	*R*_int_ = 0.012
Rotation method data acquisition using ω and phi scans	θ_max_ = 26.4°, θ_min_ = 2.7°
Absorption correction: multi-scan (*CrysAlis RED*; Oxford Diffraction, 2009)	*h* = −10→7
*T*_min_ = 0.855, *T*_max_ = 0.906	*k* = −29→20
6106 measured reflections	*l* = −7→10

### Refinement

**Table:**

Refinement on *F*^2^	Primary atom site location: structure-invariant direct methods
Least-squares matrix: full	Secondary atom site location: difference Fourier map
*R*[*F*^2^ > 2σ(*F*^2^)] = 0.058	Hydrogen site location: inferred from neighbouring sites
*wR*(*F*^2^) = 0.153	H atoms treated by a mixture of independent and constrained refinement
*S* = 1.07	*w* = 1/[σ^2^(*F*_o_^2^) + (0.0583*P*)^2^ + 1.5332*P*] where *P* = (*F*_o_^2^ + 2*F*_c_^2^)/3
3012 reflections	(Δ/σ)_max_ = 0.009
184 parameters	Δρ_max_ = 0.43 e Å^−^^3^
19 restraints	Δρ_min_ = −0.54 e Å^−^^3^

### Special details

**Table:**

Experimental. CrysAlis RED (Oxford Diffraction, 2009) Empirical absorption correction using spherical harmonics, implemented in SCALE3 ABSPACK scaling algorithm.
Geometry. All esds (except the esd in the dihedral angle between two l.s. planes) are estimated using the full covariance matrix. The cell esds are taken into account individually in the estimation of esds in distances, angles and torsion angles; correlations between esds in cell parameters are only used when they are defined by crystal symmetry. An approximate (isotropic) treatment of cell esds is used for estimating esds involving l.s. planes.
Refinement. Refinement of *F*^2^ against ALL reflections. The weighted *R*-factor wR and goodness of fit *S* are based on *F*^2^, conventional *R*-factors *R* are based on *F*, with *F* set to zero for negative *F*^2^. The threshold expression of *F*^2^ > σ(*F*^2^) is used only for calculating *R*-factors(gt) etc. and is not relevant to the choice of reflections for refinement. *R*-factors based on *F*^2^ are statistically about twice as large as those based on *F*, and *R*- factors based on ALL data will be even larger.

### Fractional atomic coordinates and isotropic or equivalent isotropic displacement parameters (Å^2^)

**Table:**

	*x*	*y*	*z*	*U*_iso_*/*U*_eq_	
C1	0.8834 (4)	0.06157 (12)	0.7360 (4)	0.0399 (6)	
C2	0.8115 (5)	0.05804 (15)	0.8744 (5)	0.0609 (9)	
C3	0.9088 (9)	0.0714 (2)	1.0333 (6)	0.1017 (18)	
H3	0.8610	0.0693	1.1268	0.122*	
C4	1.0737 (10)	0.0875 (3)	1.0534 (7)	0.112 (2)	
H4	1.1389	0.0957	1.1614	0.135*	
C5	1.1463 (6)	0.0921 (2)	0.9173 (7)	0.0901 (15)	
H5	1.2589	0.1042	0.9325	0.108*	
C6	1.0509 (4)	0.07867 (14)	0.7569 (5)	0.0564 (8)	
H6	1.0995	0.0812	0.6639	0.068*	
C7	0.6201 (4)	0.14562 (15)	0.4825 (5)	0.0553 (8)	
C8	0.4601 (4)	0.17935 (14)	0.4640 (5)	0.0525 (8)	
C9	0.4691 (6)	0.23742 (19)	0.4380 (8)	0.1039 (18)	
H9	0.5712	0.2538	0.4266	0.125*	
C10	0.3274 (6)	0.27170 (19)	0.4289 (9)	0.113 (2)	
H10	0.3359	0.3110	0.4108	0.136*	
C11	0.1739 (5)	0.24948 (16)	0.4456 (6)	0.0738 (12)	
C12	0.1647 (4)	0.19155 (15)	0.4642 (6)	0.0667 (10)	
H12	0.0615	0.1750	0.4714	0.080*	
C13	0.3054 (4)	0.15666 (14)	0.4729 (5)	0.0581 (9)	
H13	0.2950	0.1171	0.4849	0.070*	
C14	0.0207 (6)	0.2878 (2)	0.4412 (8)	0.1062 (18)	
H14A	−0.0105	0.3071	0.3338	0.127*	
H14B	0.0489	0.3159	0.5302	0.127*	
H14C	−0.0733	0.2648	0.4570	0.127*	
N1	0.6050 (3)	0.08618 (12)	0.4850 (4)	0.0524 (7)	
H1N	0.511 (3)	0.0699 (15)	0.482 (5)	0.063*	
O1	0.8805 (3)	0.05469 (13)	0.4188 (3)	0.0701 (7)	
O2	0.7012 (3)	−0.01317 (10)	0.5312 (4)	0.0727 (8)	
O3	0.7585 (3)	0.16727 (12)	0.4989 (5)	0.0902 (10)	
Cl1	0.60112 (17)	0.03731 (6)	0.85557 (19)	0.1056 (5)	
S1	0.77166 (9)	0.04311 (3)	0.52941 (10)	0.0470 (2)	

### Atomic displacement parameters (Å^2^)

**Table:**

	*U*^11^	*U*^22^	*U*^33^	*U*^12^	*U*^13^	*U*^23^
C1	0.0441 (15)	0.0354 (14)	0.0400 (15)	0.0042 (12)	0.0098 (12)	0.0020 (12)
C2	0.083 (2)	0.0542 (19)	0.053 (2)	0.0150 (18)	0.0314 (18)	0.0112 (16)
C3	0.165 (6)	0.097 (4)	0.047 (2)	0.046 (4)	0.034 (3)	0.008 (2)
C4	0.140 (5)	0.105 (4)	0.067 (3)	0.035 (4)	−0.027 (3)	−0.023 (3)
C5	0.072 (3)	0.077 (3)	0.098 (4)	−0.001 (2)	−0.027 (3)	−0.013 (3)
C6	0.0454 (17)	0.0522 (19)	0.067 (2)	−0.0015 (14)	0.0028 (15)	0.0024 (16)
C7	0.0408 (17)	0.0527 (19)	0.072 (2)	0.0017 (14)	0.0127 (15)	0.0123 (16)
C8	0.0416 (16)	0.0453 (17)	0.070 (2)	0.0008 (13)	0.0113 (15)	0.0130 (15)
C9	0.062 (2)	0.058 (2)	0.194 (5)	0.0004 (19)	0.035 (3)	0.035 (3)
C10	0.079 (3)	0.049 (2)	0.214 (6)	0.010 (2)	0.040 (3)	0.038 (3)
C11	0.056 (2)	0.048 (2)	0.117 (4)	0.0122 (16)	0.019 (2)	0.020 (2)
C12	0.0449 (17)	0.050 (2)	0.107 (3)	0.0012 (15)	0.0212 (19)	0.007 (2)
C13	0.0466 (17)	0.0397 (17)	0.089 (3)	−0.0005 (14)	0.0184 (17)	0.0054 (16)
C14	0.079 (3)	0.070 (3)	0.174 (6)	0.030 (2)	0.037 (3)	0.027 (3)
N1	0.0356 (13)	0.0464 (15)	0.0708 (18)	0.0023 (11)	0.0038 (12)	−0.0026 (13)
O1	0.0647 (15)	0.106 (2)	0.0433 (13)	0.0244 (14)	0.0207 (11)	0.0014 (13)
O2	0.0460 (13)	0.0452 (14)	0.118 (2)	0.0032 (10)	0.0014 (13)	−0.0245 (14)
O3	0.0441 (14)	0.0663 (17)	0.163 (3)	−0.0014 (12)	0.0291 (16)	0.0272 (18)
Cl1	0.1019 (9)	0.1066 (9)	0.1379 (12)	0.0130 (7)	0.0877 (9)	0.0378 (8)
S1	0.0388 (4)	0.0499 (4)	0.0500 (4)	0.0057 (3)	0.0059 (3)	−0.0087 (3)

### Geometric parameters (Å, °)

**Table:**

C1—C6	1.378 (4)	C9—C10	1.379 (6)
C1—C2	1.381 (4)	C9—H9	0.9300
C1—S1	1.761 (3)	C10—C11	1.376 (6)
C2—C3	1.379 (7)	C10—H10	0.9300
C2—Cl1	1.734 (4)	C11—C12	1.357 (5)
C3—C4	1.353 (8)	C11—C14	1.515 (5)
C3—H3	0.9300	C12—C13	1.382 (5)
C4—C5	1.370 (8)	C12—H12	0.9300
C4—H4	0.9300	C13—H13	0.9300
C5—C6	1.384 (6)	C14—H14A	0.9600
C5—H5	0.9300	C14—H14B	0.9600
C6—H6	0.9300	C14—H14C	0.9600
C7—O3	1.201 (4)	N1—S1	1.645 (3)
C7—N1	1.385 (4)	N1—H1N	0.840 (19)
C7—C8	1.485 (4)	O1—S1	1.420 (3)
C8—C9	1.369 (5)	O2—S1	1.426 (3)
C8—C13	1.370 (4)		
			
C6—C1—C2	120.1 (3)	C11—C10—C9	122.0 (4)
C6—C1—S1	117.1 (2)	C11—C10—H10	119.0
C2—C1—S1	122.8 (3)	C9—C10—H10	119.0
C3—C2—C1	119.5 (4)	C12—C11—C10	117.1 (4)
C3—C2—Cl1	118.3 (4)	C12—C11—C14	121.3 (4)
C1—C2—Cl1	122.1 (3)	C10—C11—C14	121.6 (4)
C4—C3—C2	120.2 (5)	C11—C12—C13	121.4 (3)
C4—C3—H3	119.9	C11—C12—H12	119.3
C2—C3—H3	119.9	C13—C12—H12	119.3
C3—C4—C5	121.1 (5)	C8—C13—C12	121.2 (3)
C3—C4—H4	119.5	C8—C13—H13	119.4
C5—C4—H4	119.5	C12—C13—H13	119.4
C4—C5—C6	119.6 (5)	C11—C14—H14A	109.5
C4—C5—H5	120.2	C11—C14—H14B	109.5
C6—C5—H5	120.2	H14A—C14—H14B	109.5
C1—C6—C5	119.5 (4)	C11—C14—H14C	109.5
C1—C6—H6	120.2	H14A—C14—H14C	109.5
C5—C6—H6	120.2	H14B—C14—H14C	109.5
O3—C7—N1	119.8 (3)	C7—N1—S1	122.6 (2)
O3—C7—C8	123.5 (3)	C7—N1—H1N	122 (3)
N1—C7—C8	116.7 (3)	S1—N1—H1N	115 (3)
C9—C8—C13	117.9 (3)	O1—S1—O2	119.06 (17)
C9—C8—C7	117.3 (3)	O1—S1—N1	109.85 (16)
C13—C8—C7	124.8 (3)	O2—S1—N1	104.63 (14)
C8—C9—C10	120.2 (4)	O1—S1—C1	107.63 (15)
C8—C9—H9	119.9	O2—S1—C1	109.23 (16)
C10—C9—H9	119.9	N1—S1—C1	105.67 (14)
			
C6—C1—C2—C3	−0.4 (5)	C9—C10—C11—C12	−2.8 (9)
S1—C1—C2—C3	177.8 (3)	C9—C10—C11—C14	177.9 (6)
C6—C1—C2—Cl1	179.5 (3)	C10—C11—C12—C13	2.5 (7)
S1—C1—C2—Cl1	−2.3 (4)	C14—C11—C12—C13	−178.2 (5)
C1—C2—C3—C4	−0.3 (7)	C9—C8—C13—C12	−3.1 (6)
Cl1—C2—C3—C4	179.8 (4)	C7—C8—C13—C12	176.1 (4)
C2—C3—C4—C5	1.3 (8)	C11—C12—C13—C8	0.4 (7)
C3—C4—C5—C6	−1.6 (8)	O3—C7—N1—S1	6.5 (5)
C2—C1—C6—C5	0.1 (5)	C8—C7—N1—S1	−171.8 (3)
S1—C1—C6—C5	−178.2 (3)	C7—N1—S1—O1	−55.4 (3)
C4—C5—C6—C1	0.9 (6)	C7—N1—S1—O2	175.7 (3)
O3—C7—C8—C9	10.5 (6)	C7—N1—S1—C1	60.4 (3)
N1—C7—C8—C9	−171.3 (4)	C6—C1—S1—O1	−4.7 (3)
O3—C7—C8—C13	−168.8 (4)	C2—C1—S1—O1	177.0 (3)
N1—C7—C8—C13	9.5 (5)	C6—C1—S1—O2	125.9 (2)
C13—C8—C9—C10	2.8 (8)	C2—C1—S1—O2	−52.4 (3)
C7—C8—C9—C10	−176.5 (5)	C6—C1—S1—N1	−122.0 (2)
C8—C9—C10—C11	0.2 (10)	C2—C1—S1—N1	59.7 (3)

### Hydrogen-bond geometry (Å, °)

**Table:**

*D*—H···*A*	*D*—H	H···*A*	*D*···*A*	*D*—H···*A*
N1—H1N···O2^i^	0.84 (2)	2.14 (2)	2.970 (4)	169 (4)

Symmetry codes: (i) −*x*+1, −*y*, −*z*+1.

## References

[bb1] Gowda, B. T., Foro, S., Suchetan, P. A. & Fuess, H. (2010*a*). *Acta Cryst.* E**66**, o747. 10.1107/S1600536810007440 PMC2984015 21580592

[bb2] Gowda, B. T., Foro, S., Suchetan, P. A. & Fuess, H. (2010*b*). *Acta Cryst.* E**66**, o794. 10.1107/S1600536810008731 PMC2984022 21580633

[bb3] Oxford Diffraction (2009). *CrysAlis CCD* and *CrysAlis RED* Oxford Diffraction Ltd, Yarnton, England.

[bb4] Sheldrick, G. M. (2008). *Acta Cryst.* A**64**, 112–122. 10.1107/S0108767307043930 18156677

[bb5] Spek, A. L. (2009). *Acta Cryst.* D**65**, 148–155. 10.1107/S090744490804362X PMC2631630 19171970

[bb6] Suchetan, P. A., Gowda, B. T., Foro, S. & Fuess, H. (2010*a*). *Acta Cryst.* E**66**, o1281. 10.1107/S1600536810015990 PMC2979549 21579380

[bb7] Suchetan, P. A., Gowda, B. T., Foro, S. & Fuess, H. (2010*b*). *Acta Cryst.* E**66**, o1292. 10.1107/S1600536810016235 PMC2979524 21579389

